# Cake Perception, Texture and Aroma Profile as Affected by Wheat Flour and Cocoa Replacement with Carob Flour

**DOI:** 10.3390/foods9111586

**Published:** 2020-11-02

**Authors:** Maria Papageorgiou, Adamantini Paraskevopoulou, Foteini Pantazi, Adriana Skendi

**Affiliations:** 1 Department of Food Science and Technology, International Hellenic University, POB 141, GR-57400 Thessaloniki, Greece; andrianaskendi@hotmail.com; 2 Laboratory of Food Chemistry and Technology, School of Chemistry, Aristotle University of Thessaloniki, 54124 Thessaloniki, Greece; pantazfa@outlook.com

**Keywords:** carob, cocoa substitution, cake, aroma, color, texture, sensory evaluation

## Abstract

Carob flour has been used in the production of a wide range of functional food formulations such as bakery goods either as a natural sweetener or food ingredient that, when roasted, exerts a chocolate/cocoa-reminiscent flavor and color. The aim of the present study was twofold; firstly to study the effect of an increasing incorporation of roasted carob flour (0–70% flour basis) on the quality and sensory attributes of a conventional cocoa cake recipe and secondly to investigate the obtained volatile fraction responsible for the aroma by means of headspace solid phase microextraction (HS-SPME) technique coupled to gas chromatography/mass spectrometry (GC/MS) while comparing it with the control, cocoa-containing cake recipe. Thirty and fifty percent carob flour incorporation rendered cakes with acceptable texture and sensory attributes, comparable to the control cake recipe containing 20% cocoa. Similarity to cocoa aroma was attributed to a great number of odor active compounds mainly belonging to aldehydes, lactones, furan/pyran derivatives, and pyrrole derivatives.

## 1. Introduction

Cake, as a popular baked dessert consumed worldwide, could represent an excellent vehicle for introducing new health-beneficial eating habits. In this direction, proteins and dietary fibers from various sources (e.g., legumes, cereal bran, pulp and peel flours from fruits and fruit pomaces remaining after juice extraction) have been used in the preparation of conventional as well as gluten-free cake formulations [1,2,3,4,5,6].

Carob flour, produced from the fruits of *Ceratonia siliqua L.* after the removal of their seeds and subsequent roasting, has gained increased interest due to its remarkable composition, which is responsible for exerting a preventive action against various diseases [7]. It is characterized by a high content of sugars (>50%, mainly sucrose), dietary fibers (~11%), minerals (Mg, Fe, P, Zn, Ca, K, Na) as well as low protein (3–4%) and lipids (0.2–0.8%) levels and significant amounts of phenolic compounds (e.g., gallic acid, hydrolysable and condensed tannins) and vitamins (e.g., E, D, C, B6, folic acid) [7,8,9]. Carob flour has been used in the production of a wide range of functional food formulations such as bakery goods, sweets, pasta and beverages, either as a natural sweetener or food ingredient. 

Moreover, its chocolate/cocoa-reminiscent flavor and color, combined with the fact that it is free of caffeine and theobromine stimulants, much cheaper than cocoa, increases fiber intake, contains less fat and possesses notable free radical scavenging activity, makes it a particularly important cocoa alternative and extender in the food industry [10]. However, despite the similarity of its aroma with that of cocoa and the reported potential use as a flavoring agent in many food applications, the literature data concerning its aroma profile are limited. According to McLeod and Forcen [11], the powdered carob bean pulp extracts obtained by applying the simultaneous steam distillation-solvent extraction technique were characterized by the presence of high levels of aliphatic acids followed by ketones, aldehydes, benzene derivatives, alcohols, esters, terpenes, furan derivatives and other compounds, while their aroma was described as “sweet, buttery, caramel, estery, slightly oily/fatty, with unpleasant sulphurous and rancid/sweaty overtones”. It was observed that the roasting process significantly affects the aroma profile of carob flours, which varies depending on the roasting degree [12]. Based on GC-MS data along with sensory observations, cocoa-like odor notes were chiefly ascribed to sugars’ thermal degradation rather than to Maillard reaction pathways. Fadel et al. [13], who compared the volatile flavor compounds of real cocoa powder with that of a low-cost cocoa substitute composed of a milled and roasted carob pods and chicory roots (2:1 *w*/*w*) blend, reported that most of the volatile compounds identified in real cocoa were also depicted in the cocoa substitute sample. Thus, carob pulp should be roasted and grinded to powder before use as a cocoa substitute [14]. 

Cocoa powder replacement by carob flour has been evaluated only for gluten-free cakes prepared with soy and banana flours [15]. A more recent study has reported the successful replacement of 10% of cocoa powder with carob flour in powder drink mixtures [16] and another one its 5% addition in soy muffins [17]. Although the recent literature reports the use of carob in cake recipes [18,19,20], the aspect of cocoa replacement in terms of aroma profile is not touched upon but rather the effect of high-level carob addition on the quality, chemical composition, sensorial characteristics of the cakes. 

Thus, our intent was not only to study the effect that the increasing incorporation of roasted carob flour (0–70%) has on the quality and sensory attributes of a conventional cocoa cake recipe, but also to investigate the obtained volatile fraction responsible of the aroma product in the best performed carob cake while comparing it with that of the control cocoa-containing cake recipe. A detailed evaluation of the developed aroma volatile fraction of pound cake with the added carob flour or cocoa was performed by means of headspace solid phase microextraction (HS-SPME) technique coupled to gas chromatography/mass spectrometry (GC/MS) analysis.

## 2. Materials and Methods 

### 2.1. Materials

Wheat flour (12.19 ± 0.06 moisture; 9.37 ± 0.18% protein; 2.05 ± 0.01% ash; 1.05 ± 0.15% fat), commercial margarine (60% fat) (Vitam soft, Upfield Europe BV, Rotterdam, Netherlands), sugar, fresh whole eggs, baking powder (Yiotis S.A., Athens, Greece), and cocoa powder were purchased from the local market. Carob flour (6.70 ± 0.09% moisture; 4.39 ± 0.06% protein; 2.10 ± 0.06% ash; 0.32 ± 0.10% fat; 33.02 ± 0.97% total dietary fiber) was a commercial organic product (roasted) (Creta Carob, Greece). Moisture and fat content were determined based on AACC methods 44-15A and 30-10, respectively, whereas proteins and ash content were determined according to the ICC official methods 105/2, 104/1 and 155, respectively. For the determination of the total dietary fiber, the procedure described in the “Total dietary fibre assay” was used (AACC Method 32-07.01), using the respective test kit provided by Megazyme International Ireland Ltd. (Wicklow, Ireland). Deionized water was used in all formulations.

### 2.2. Cake Preparation and Measurements

A Madeira-type cake was prepared by replacing cocoa powder and, partly, wheat flour with carob flour. The recipes consisted of wheat flour, sugar, margarine, eggs, cocoa and carob flour, as reported in Table 1. The control sample contained wheat flour and cocoa powder, in total, 75 g (100% flour basis).

Sugar was first mixed with margarine for seven minutes at the maximum speed (3rd) of a hand mixer (Kenwood, model HM52, with three speed selection) till formation of a creamy mixture, and then the whole eggs were added and mixed for one more minute. Following this, wheat flour with cocoa or carob flour was added, and the mixture was mixed for three to four minutes. Margarine and eggs were equilibrated at room temperature before use. The determination of cake batter density was performed in a pre-weighted 100 mL cylinder according to Paraskevopoulou et al. [21]. The cylinder was filled with cake batter and reweighted. The density was the ratio of the weight of the batter to the respective weight of deionized water. The cake batter (300 g, as reported in Table 1) was placed in greased aluminum pans (11.15 × 8.65 × 4 cm) and baked at 180 °C for 40 min in a preheated oven (Neff, Germany). At least three cakes per recipe were prepared.

After baking and cooling at room temperature, the cakes were removed from their pans. The following quality characteristics were assessed on the day of baking: volume, cake yield, crust and crumb color, and texture of the crumb. Volume was measured with rapeseed method, whereas cake yield was determined as reported in Paraskevopoulou et al. [21]. Color CIE parameters (L* (dark–light, 0/100), a* (green–red, −/+), b* (blue–yellow, −/+) scale) were measured with Chromameter (Dr. Lange, Germany) on the crust and the crumb of the cakes. For the texture analysis, each cake was sliced in 2 cm thick slices and, from the centre of each slice, a piece of crumb with 2.2 cm diameter was cut by using a metallic cylinder cutter. The crumbs were immediately double-compressed on a TA-XT2 Texture analyzer (Stable Micro Systems Ltd., Surrey, UK) at 50% compression, speed 1 mm/s using a 100 mm aluminum probe. The following parameters were measured: hardness, springiness, adhesiveness, and cohesiveness. At least three replicates for each recipe were analyzed.

### 2.3. Sensory Test

The sensory evaluation of the cakes was performed 24 h after baking by 20 trained panelists, aged 30–62 years (11 men and nine women) at the sensory laboratory of the department of the Food Science and Technology of the International Hellenic University. The laboratory is equipped with individual compartments and the tests were performed under white light at room temperature. The panelists were offered 1 cm thick, freshly cut cake slices (without the crust), coded with random three-digit numbers. The samples were presented to each panelist in a balanced incomplete block design (parameters: *t* = 5 treatments, *k* = 3 treatments per panelist, *b* = 20 panelists, *n* = 12 replicates per treatment, *λ* = 2 pairs of similar treatments in the design).

The cakes were evaluated for their color, alveolar structure (porosity), aroma, sweetness, softness, crumbliness, and overall acceptability. Three slices from different cake recipes were served on white plastic plates together with detailed information on the procedure and advice to rinse their palate with water to avoid residual effects. The panelists rated arbitrary the intensity of each feature by placing a mark on a linear scale of 15 cm in length with marks at the ends and in the middle of the scale [22]. The left end of the scale corresponded to “none”, while the right end of the scale represented a large amount, or a “very” level. The middle referred to a moderate amount. The sensory test protocols were in line with the provisions of the Bioethics’ Committee of the International Hellenic University.

### 2.4. Volatile Compounds Analysis

A cake slice, cut from the center of the loaf after cooling, was immediately frozen with liquid nitrogen, crumbled with a mortar and pestle and kept in the freezer (−30 °C) till analysis. Volatile compound isolation was carried out by HS-SPME (Supelco, Bellefonte, PA, USA) with the following conditions: 2 g of frozen cake sample, 1 mL of NaCl solution (200 g/L) and 1 μL of 4-methyl-2-pentanol as internal standard (1.8 mg/mL ethanolic solution) were blended in a 15 mL glass vial containing a magnetic stirrer. The sample vial was closely capped with a PTFE-silicon stopper and equilibrated at 60 °C for 30 min. Then, a 75 μm CAR/PDMS fiber (Supelco, Bellefonte, PA, USA) was placed in the headspace to extract the volatiles for 60 min under continuous heating and stirring. After extraction, the fiber was immediately desorbed into the injection port of an Agilent 6890N gas chromatograph equipped with MSD 5973 mass spectrometer (Palo Alto, CA, USA) at 250 °C for 5 min in splitless mode. A HP-FFAP capillary column (30 m × 0.32 mm i.d. × 0.25 μm film thickness; Agilent Technologies, Wilmington, DE, USA) was employed for separating the volatile compounds of the cakes. The GC-MS temperature conditions were the same as in our previous report [23]. Helium was used as the carrier gas at a flow rate of 1.8 mL/min. Volatile identification was based on the comparison of the retention indices (RI), calculated by the retention time of a C7–C26 n-alkane series (Supelco, Bellefonte, PA, USA) under the same chromatographic conditions, to those of available standards or reported in the literature (tentatively), as well as on the matching of their mass spectra with those in the NIST library (Version 2.0g, 2011, Gaithersburg, MD, USA). Semi-quantitative data of the identified compounds were measured from the characteristic ion peak areas with regard to the peak area of the internal standard assuming a response factor equal to one. Concentrations were expressed as mg 4-methyl-2-pentanol equivalents per kg of sample. Odor activity value (OAV) was calculated by dividing the concentration of a compound by its odor threshold from aqueous solution. Compounds with OAVs > 1 were considered as aroma contributors.

### 2.5. Statistical Analysis

All the results were expressed as mean ± standard deviation (SD). The statistical analysis was conducted by one-way analysis of variance (ANOVA) to assess differences among the treatments (*p* < 0.05) followed by Tukey’s pairwise comparison of means using Minitab^©^ Statistical Software v 18.1 (Minitab Inc., State College, PA., USA.). The correlation between the sensory attributes was also investigated by principal component analysis (PCA) using the SPSS Statistics 25.0 software (SPSS Inc., Chicago, IL, USA). Eigenvalues higher than 1.0 were used. Moreover, the Independent Samples *t*-Test (*p* < 0.05) was used to detect differences among the means of the volatile compounds using SPSS package.

## 3. Results

### 3.1. Effect of Carob Flour in the Physical Properties of Batter and Cake

The effect of carob flour incorporation on cake batter density, yield and specific volume is shown in Table 2. Batter density is linked to the ability of the mixture to entrap and hold air during mixing. Thus, a decrease in the density is related to higher final volume and soft texture. The density of the batter was increased by increasing the level of substitution. Nevertheless, only a 70% addition level produced a significant increase of the cake batter density. This is contrary to the findings of Paraskevopoulou et al. [21], who used a maize-milling by-product to substitute 30% of the wheat flour in a high-in-fiber cake and to those of Gómez et al. [24] who used wholegrain cereal flours. Most likely by increasing the substitution level, the membrane that holds the air bubbles is becoming less elastic due to the non-protein constituents of the carob flour that disrupt the gluten structure thus decreasing the ability of batter to hold the air. Nevertheless, at substitution levels lower than 70% flour basis, carob cake batters exhibited similar densities with that of the control (cake with 20% cocoa). On the other hand, a significant decrease was observed in the cake-specific volume with increasing levels of wheat flour substitution. It seems that the weakened batter matrix due to the incorporation of the carob flour cannot withstand air bubble expansion during baking. The addition of carob flour at the highest level of substitution produced significantly lower cake yield compared to the control cocoa cake, suggesting that the batter structure was not able to retain moisture. This is contrary to the findings of Rosa et al. [15]. Differences could be due to the different cake composition in the two studies. The maximal level of carob cake that they used in their recipe was 25%. Moreover, instead of wheat flour, they used soy flour and banana flour, and besides the eggs, margarine and sugar, they used milk to moisten their cake and yeast as a leavening agent. The higher level of proteins in their recipe could have helped in retaining more moisture.

**Figure 1:**
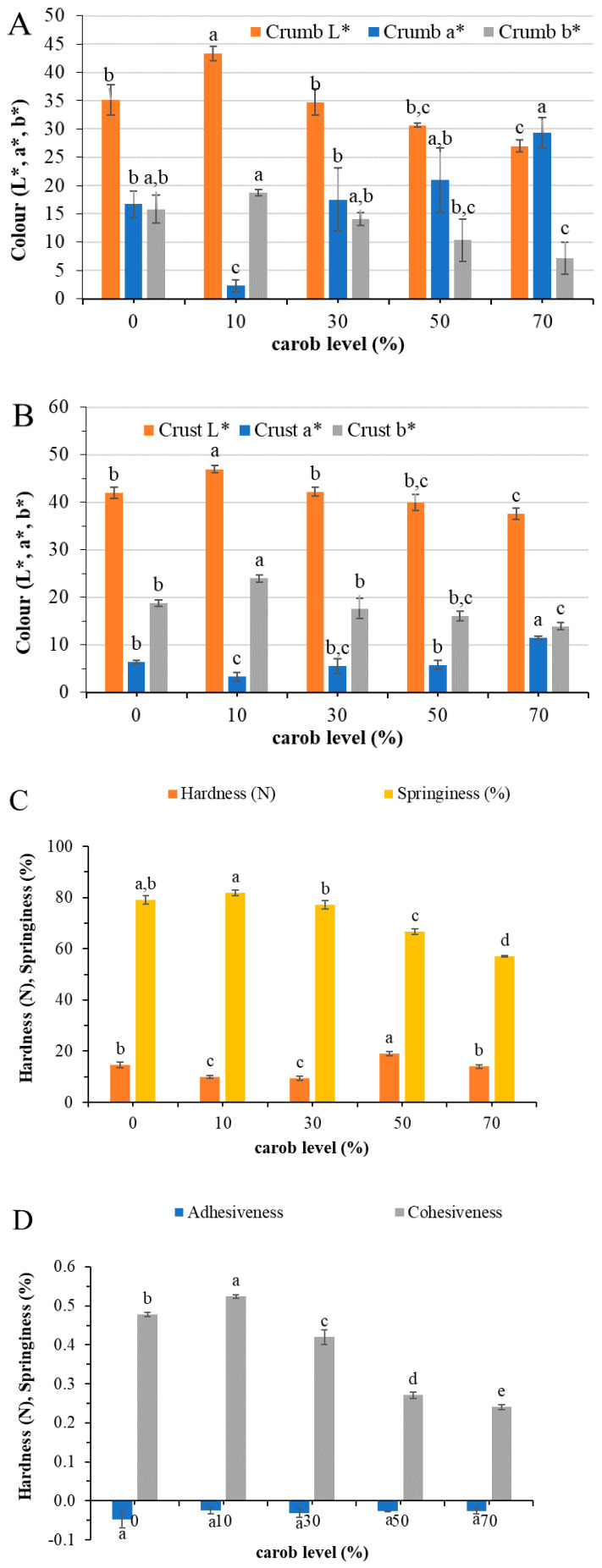
Crumb (**A**) and crust (**B**) color, as well as texture parameters (**C**,**D**) of control cake ((0% carob, 15% cocoa (f.b.)) and carob cakes prepared with different levels of carob flour. Data represent means ± SD (*n* = 3). Means that do not share a letter are significantly different (*p* < 0.05) according to Tukey’s pairwise comparison of means.

Carob flour addition up to 10% flour basis resulted in cakes with lighter crumb color than the control (Figure 1A). At substitution levels of 30 and 50% carob flour, the final products exhibited a similar brightness with the control, while further increase rendered significantly darker cakes (*p* < 0.05). On the other hand, the progressive increase in the level of carob flour produced cakes with less yellow and more intense red hue compared to the control cocoa cake. Similarly, Rosa et al. [15] observed an increase in the a* and a decrease in the chromaticity coordinate b* with the increased substitution of cocoa powder with carob flour. In parallel to the crumb, the crust of the cake with 10% carob flour was significantly lighter, less red and more yellow than the control cake (Figure 1B). Increasing the level of substitution up to 50% resulted in cakes with similar color parameters (L*, a*, b*) to the control, whereas, upon further increase, the cakes became darker and more reddish. Pawłowska et al. [17], in their study, reported that the muffins prepared with carob flour showed a lighter crumb than the respective with the same amount of cocoa (5%), but they did not differ in the color of the crust. It is noteworthy that these muffins contained only 6% wheat flour; the other floury ingredients being soybeans (25.5%) and flaxseeds (6.5%) with distinctive color parameters of their own. In the study of Rosa et al. [15], replacement of cocoa at the same level as carob flour resulted in cakes with darker crust surface. 

Looking at the textural attributes, it was observed (Figure 1C,D) that only the incorporation level of 50% flour basis rendered cakes with a significantly (*p* < 0.05) harder crumb than the control cocoa cake. At addition levels of 10 and 30% carob flour, the hardness of the crumb was significantly reduced, with no significant changes in the springiness values when compared to the control cake. These cakes have also shown a similar specific volume. By increasing the carob level above 30% flour basis, a significant decrease of the springiness and cohesiveness was noticed. No changes were observed in the adhesiveness values among all the tested samples.

### 3.2. Effect of Carob Flour in the Sensory Analysis

In Table 3, the mean values assigned to the attributes of the cakes that were tested by the panelists are reported. On the basis of the one-way ANOVA applied for all the attributes tested by the panelists, significant differences (*p* < 0.05) were observed only for the color, sweetness and softness. Panelist ratings of cakes with regard to color were in agreement with the measured values by the chromameter. They evaluated the color of cakes with 30 and 50% carob flour as similar to the cake with 20% cocoa (control). Regarding sweetness, a progressive increase was observed in the perceived sweetness of the cakes with increasing levels of carob flours. This was expected due to the sugar content of the carob flour, on average 40%, as derived from the literature [8]. However, only the cake with 70% carob flour was significantly different from the control. All carob-containing cakes up to a 30% substitution level were considered to have similar softness with the control cake, although with increasing level of substitution beyond this level, the panelists perceived the resulting cakes as less soft. It seems that panelists appreciated all the carob-containing cakes, since their score in overall acceptability was statistically similar to that of the control (Table 3). In absolute values, the cake with 30% carob flour was on the top of the rank scoring of 10.4 in overall acceptability. However, the cake with 50% carob flour gained the highest score for overall acceptability from ~42% of the 12 panelists that were presented with the above sample (data not shown); the above percentage of panelists was the same for the control cake, while for the cakes with 30% and 70% carob it was ~33%. The corresponding percentage for the cake with 10% carob was only ~17%. A standard acceptability test with 100 consumers should be performed to confirm sensory acceptability results. The intensity of the aroma did not differ significantly among samples, while the sample with 50% carob flour scored highest and that with 10% lowest. It should be noted that aroma intensity was not evaluated in terms of cocoa aroma intensity but as the aroma of the baked product. 

**Figure 2:**
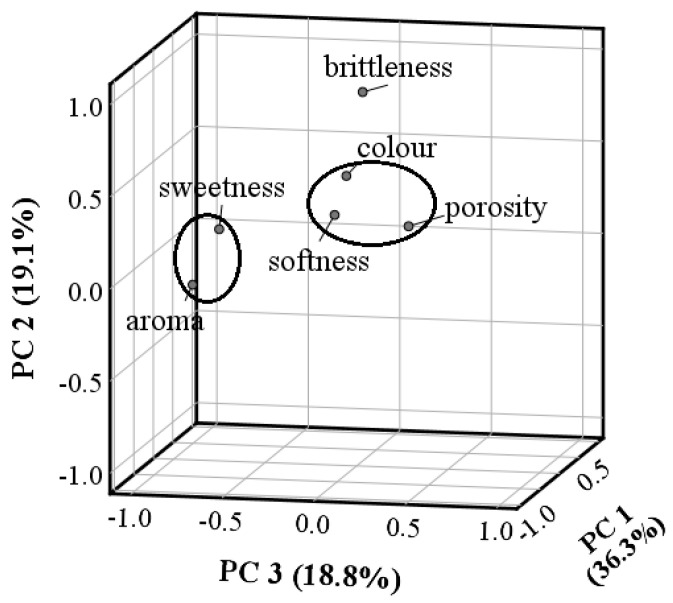
3D Component plot in rotated space of correlation coefficients between sensory attributes (all the three components account for 74.1% of all the variation.

PCA analysis was conducted in order to evaluate the relationship between the sensory attributes of cakes assessed by trained panelists (Figure 2, Table 4). In Figure 2, the 3D PCA plot of correlation coefficients is reported, whereas in Table 4 the correlation coefficients of the attributes tested with the three major components are reported, as well as the communalities that explain the proportion of the variance for each attribute that can be explained by the components. The three axes explained 74.1% of the total variation. Component 1 (PC1) represents 36.3% of the total variance and is positively related to color and porosity and negatively to the softness, whereas component 2 (PC2) is correlated positively to the brittleness. Indeed, the three attributes are linked, since more porous structures are perceived as darker in color and softer. Component 3 (PC3) accounts alone for 18.8% of the total variance and is negatively correlated to the aroma and sweetness. Both these attributes are linked since, depending on the sweetness, one can perceive the aroma as more or less intense.

### 3.3. GC-MS Analysis

Since the cake with 30% carob flour gained the highest score for overall acceptability, while it also exhibited similar aroma intensity and color with the control cake, it was decided to proceed with the identification of the aroma profile of these two samples for comparison purposes. A total of 113 headspace volatile compounds isolated by SPME were identified in the studied cakes, including 18 aldehydes, 17 alcohols, 12 ketones, 20 nitrogen-containing compounds (16 pyrazine and 3 pyrrole derivatives), 16 carbonic acids, 8 furan derivatives, 3 pyran derivatives, 10 lactones, 6 esters, 2 sulphur compounds and 2 terpene compounds, as shown in Table 5. As was revealed, a large number of compounds were developed during baking (i.e., 102 and 98 for carob and cocoa-containing cakes, respectively), 86 of which were identical in both samples. The total concentration of volatile compounds differed and was greatly affected by the replacement of wheat flour with carob flour (~35 mg/kg) compared to the cocoa-containing cake (~22 mg/kg). Moreover, in an attempt to clarify the aroma similarity between the two cakes perceived by the assessors and in order to gain an insight into the relative contribution of each volatile compound to the overall aroma, the odor activity values were calculated. Forty-nine (49) compounds were found to be present at concentrations above their odor thresholds (i.e., OAV > 1) and are presented in bold in Table 5.

Most of the identified carbonyl compounds have been already identified in cakes [25,26], and their presence has mostly been ascribed to the involvement of lipids in the raw materials as well as Maillard and Strecker degradation reactions. Some of them are derived from the auto-oxidation of unsaturated fatty acids, i.e., (*E*)-2-hexenal, (*E*)-2-heptenal, (*E*)-2-octenal, (*E*)-2-nonenal, (*E,E*)-2,4-heptadienal and (*E,E*)-2,4-decadienal are formed by the oxidation of linoleic and linolenic acids; octanal and nonanal are formed by the oxidation of oleic acid [27]. The Strecker aldehyde 3-methyl-butanal (“malty, cocoa-like”) and hexanal (“grass, fatty”) were found to be present at high concentrations in both cakes (*p* > 0.05). Additionally, regarding benzaldehyde and phenylacetaldehyde, that may provide a “nutty and honey-like” smell, there were significant differences in their content between the two cakes, with cocoa cakes having the highest total concentration. Both compounds have been previously detected among the compounds of high influence on chocolate and cocoa, respectively [28]. What is worth emphasizing in this place is that almost all detected aldehydes could be regarded as playing an important role in the characteristic cake aroma. As shown in Table 5, the summation of OAVs revealed that aldehydes occupied 93.2 and 91.8% of the total OAVs in the carob- and cocoa-containing cakes, respectively. Among them, (*E,E*)-2,4-decadienal possessed the highest OAVs (3098.6, 2472.9) followed by 3-methyl-butanal, (*E,Z*)-2,4-decadienal, hexanal, octanal and nonanal. The high amount of (*E,E*)-2,4-decadienal in carob cakes is probably due to the decomposition of linoleic acid, one of the main fatty acids of carob flour. Notably, 3-methyl-butanal exhibited similar OAVs in both cakes (1786.8 and 1841.4, respectively) and could be suggested to be an important contributor to their aroma profile. With respect to ketones, carob cake was the richest most likely due to amino acid Strecker and lipid degradation reactions. Among the ketones detected, 1-hydroxy-2-propanone, imparting “caramel-like” notes, was detected only in carob cakes, while eight ketones were found at concentrations above their respective odor thresholds (OAV > 1). Among them, 1-penten-3-one (“pungent, spicy”), 2-nonanone (“flowery, fatty”), 2-undecanone (“fruity, fresh”) and 2,3-pentanedione (“caramel, sweet, fruity, buttery”) are more important sensorially than others because of their very low threshold value. The other ketones (2-heptanone, 2-octanone, 6-methyl-5-hepten-2-one and 3,5-octadien-2-one) presented the highest amount in carob samples, probably due to their presence in a volatile fraction of the carob flour [11].

Alcohols, being common volatiles in cakes and breads as well, were the most plentiful group for all cake samples. Ethyl alcohol, although present at high levels, did not reach its perception threshold in any type of cake. Based on the obtained results, eight exceeded their odor threshold, with 1-octen-3-ol (“earthy, green”) presenting the highest OAV, followed by 1-hexanol and 1-pentanol, while 2-methoxy-4-vinyl-phenol may provide a smell of herbs to both of them.

The carbonic acid fraction was the second dominant group of volatiles in carob-containing cakes. Sixteen compounds were detected, followed by 12 compounds in the cocoa-containing sample, which, however, was found to contain total carbonic acids at a much lower concentration (8.182 vs. 1.236 mg/kg). More than 75% of the total acid content in carob cake was due to the presence of hexanoic acid (OAV = 2.0), followed by 2-methyl-propanoic (OAV = 4.5) and butanoic acid (OAV = 2.3). An amount is likely to be formed by the oxidation of saturated volatile aldehydes, but the greatest percentage originates from carob flour. According to the literature data, the volatile fraction of carob flour is represented by a very high level (i.e., 77.5%) of aliphatic acids, the major contributors being methylpropanoic acid (45.0%), hexanoic acid (19.0%), 2-methylbutanoic acid (8.0%), butanoic acid (5.0%) and others (e.g., pentanoic, heptanoic, octanoic, benzoic) [11,29]. In general, these acids are described as having “cheesy, fatty, sweaty” odor notes. Besides this, the presence of acids justifies the presence of esters that were detected in both cake samples and which are coming from the esterification reaction of short-chain alcohols with a large amount of free fatty acids, that are present in the raw materials. All of them are described as having “fruity” and “sweet” odors, such as pineapple and honey.

The compounds that are primarily responsible for the characteristic aroma of chocolate are nitrogen- and oxygen-containing molecules, such as pyrazine, pyrrole, furan, and pyran derivatives, mainly generated during sugars’ thermal degradation and Maillard reactions [13]. These are well known key odorants in heat-processed food products, particularly when treatment involves baking (e.g., cake, bread) or roasting (e.g., coffee, cocoa, nuts), and are considered to be among the most important aroma contributors, providing them with a rich “roasted” flavor. They are generally referred to as “nutty-like”, “cocoa-like” and “sweet/caramel-like” [13,25,30,31]. More specifically, almost all the pyrazine derivatives were detected in the cocoa-containing cakes (15 compounds, 1.91 mg/kg) instead of 10 compounds (0.61 mg/kg) in the carob cakes owing, probably, to the variation in protein and amino acid concentrations between raw materials. Most of them (e.g., methylpyrazine, dimethylpyrazines, trimethylpyrazine, 2-ethyl-6-methylpyrazine) have been previously identified in cocoa and cocoa-containing products. Nevertheless, given their relatively high odor thresholds, only five of them possessed OAVs >1, thereby having an impact on cakes’ aroma profile. Between them, two were common in both samples. More specifically, 3-ethyl-2,5-dimethylpyrazine, formed by the reaction of glucose with aspartic acid, was detected in both carob (OAV = 64.5) and cocoa (OAV = 218.8) cakes in concentrations exceeding its odor threshold and could be considered as one of the most significant contributors to the “cocoa, coffee, roasted nutty”-reminiscent smell of the products. Additionally, 2-methoxy-3-methyl pyrazine was identified between the most abundant pyrazine derivatives in cakes’ volatiles (OAV ~42), giving an “almond, earthy, roasted” note. 

Other potent aroma compounds in the studied cakes were furan derivatives, associated with “caramel” odor notes. Eight compounds were identified, accounting for the 20% of the volatile fraction of carob cakes. Among them, furfural, 2-acetyl-furan (“sweet, cocoa, coffee”) and 5-hydroxymethylfurfural (“buttery, caramel-like”) were detected at a 10-fold concentration in the carob cake in comparison to cocoa cake, while 5-acetyl-dihydro-2(3H)-furanone (“fruity”) was found only in this sample. Furfural, being widely reported to contribute a “sweet, almond-like” odor, was present at a concentration over its detection threshold (OAV = 1.6) and thus, probably, perceptible in the carob cake. Otherwise, 2-pentyl furan (“green, earthy, beany”), a product of the oxidative degradation of linolenic acid, was found to exert an impact on both cakes’ aroma. Three pyran derivatives, i.e., maltol, 5-hydroxymaltol and 2,3-dihydro-3,5-dihydroxy-6-methyl-4H-pyran-4-one, and three pyrrole derivatives, i.e., 1-ethyl-1H-pyrrole-2-carboxaldehyde, 2-acetylpyrrole and 2-formyl pyrrole, were also identified in the studied cakes, notably predominating in the carob-flour-containing one (*p* < 0.05). The Maillard reaction product 1-ethyl-1H-pyrrole-2-carboxaldehyde, a compound found among the carob flour’s aroma contributors, existed only in carob cake and may be partly responsible for “roasted, burnt, smoky” odor (odor threshold not available) [11,32]. The much higher sugar content of carob flour (40–50%) than that of the cocoa powder (2–4%) or wheat flour (~1%) may explain the high concentrations of furan/pyran as well as pyrrole derivatives observed in the case of carob cakes [33,34].

Ten lactones, resulting from the intramolecular esterification of their corresponding hydroxycarboxylic acids, were also detected, i.e., 10 in the carob (0.53 mg/kg) and seven in the cocoa cakes (0.229 mg/kg). Their abundance in the carob cake at concentrations over their odor thresholds, e.g., *γ*-hexalactone (OAV = 1.4), *γ*-octalactone (OAV = 3.8), *γ*-nonalactone (OAV = 3.5), *δ*-decalactone (OAV = 4.1), could be attributed either to the high concentration of their precursors or to their presence in the carob flour, as has been previously reported by Cantalejo [32]. Their odor notes, mostly similar to “cream, caramel, fruits and butter”, are likely to contribute to the characteristic aroma of the carob cakes, reminiscent of that of cocoa ones [31].

In the group of sulphur-containing compounds, two constituents, dimethyl disulphide and methional, were identified. They are typical of carob flour’s flavor profile [11,32]. Both have been detected exclusively in carob cakes presenting OAVs >1 (10.6 and 179, respectively). Dimethyl disulphide has been previously reported to give rise, in the presence of aldehydes such as 3-methylbutanal, to a cocoa-like aroma [11,35].

## 4. Conclusions

Thirty and fifty percent carob flour incorporation rendered cakes with acceptable texture and sensory attributes, comparable to the control cake recipe containing 20% cocoa. Batter density and cake yield were only affected at a 70% incorporation level. Cake specific volume was negatively affected by the incorporation of the high content of dietary fibers in the raw material added. PCA analysis of the dataset explained the 74.1% of the total variation, revealing a high association between some sensory parameters. A close positive relationship was observed between the color and porosity, whereas the softness was inversely associated with those parameters. On the other hand, aroma and sweetness have a high relationship between them.

The cake with 30% carob flour gained the highest score for overall acceptability while its aroma was perceived as similar to that of the cocoa-containing cake. A total of 113 headspace volatile compounds isolated by SPME were identified in the studied cakes. As revealed by GS-MS analysis, it could be concluded that the cocoa-reminiscent aroma of carob flour-containing cake could mainly be attributed to the similarities observed with the aroma profile of cocoa cake as regards the presence of aldehydes, lactones, furan/pyran derivatives, and pyrrole derivatives.

## Figures and Tables

**Table 1:** Cake recipe.

Recipe	Wheat Flour (g)	Carob Flour (g)	Margarine (g)	Sugar (g)	Egg (g)	Cocoa (g)
0 (control)	60	-	75	75	75	15
10	67.5	7.5	75	75	75	-
30	52.5	22.5	75	75	75	-
50	37.5	37.5	75	75	75	-
70	22.5	52.5	75	75	75	-

**Table 2:** Physical properties of batters and cakes.

Carob Flour Level (%)	Cake Batter Density (g/cm^3^)	Cake Specific Volume (mL/g)	Cake Yield (%)
0	0.83 ± 0.01 ^b^	2.22 ± 0.12 ^a^	8.14 ± 0.40 ^a^
10	0.78 ± 0.03 ^b^	2.01 ± 0.08 ^ab^	7.10 ±0.32 ^ab^
30	0.79 ± 0.01 ^b^	1.93 ± 0.00 ^abc^	7.37 ± 0.40 ^ab^
50	0.80 ± 0.03 ^b^	1.78 ± 0.10 ^bc^	7.45 ± 0.15 ^ab^
70	1.30 ± 0.03 ^a^	1.66 ± 0.02 ^c^	6.31 ± 0.12 ^b^

Data represent means ± standard deviation (SD, *n* = 2). Means that do not share a letter are significantly different (*p* < 0.05) according to Tukey’s pairwise comparison of means.

**Table 3:** Influence of the carob flour substitution level (0, 10, 30, 50 and 70%) on the sensory scores of different attributes assessed by panelists for the respective cakes.

Carob Flour Level (%)	Color	Porosity	Aroma	Sweetness	Softness	Brittleness	Overall Acceptability
0	7.8 ± 3.3 ^b^	7.5 ± 2.9 ^a^	7.5 ± 3.1 ^a^	6.2 ± 3.0 ^b^	8.9 ± 1.8 ^ab^	8.8 ± 2.8 ^a^	8.9 ± 3.9 ^a^
10	3.0 ± 2.7 ^c^	7.9 ± 2.7 ^a^	6.3 ± 3.6 ^a^	7.0 ± 3.1 ^b^	10.4 ± 3.2 ^a^	7.1 ± 3.7 ^a^	9.1 ± 3.0 ^a^
30	7.9 ± 3.4 ^b^	9.2 ± 3.4 ^a^	7.4 ± 3.9 ^a^	8.4 ± 2.3 ^ab^	9.7 ± 2.7 ^ab^	7.2 ± 2.5 ^a^	10.4 ± 2.9 ^a^
50	10.5 ± 2.5 ^ab^	11.0 ± 2.5 ^a^	8.5 ± 3.9 ^a^	9.1 ± 3.0 ^ab^	6.9 ± 3.1 ^b^	5.7 ± 3.4 ^a^	9.7 ± 2.5 ^a^
70	12.6 ± 1.2 ^a^	11.1 ± 2.3 ^a^	7.9 ± 3.1 ^a^	10.8 ± 3.3 ^a^	6.8 ± 3.5 ^b^	8.7 ± 4.0 ^a^	8.5 ± 4.0 ^a^

Values represent the intensity of each feature on a linear scale of 15 cm in length (from “none” to “very”). Data are represented as means ± SD (*n* = 12). Means that do not share a letter are significantly different (*p* < 0.05) according to Tukey’s pairwise comparison of means.

**Table 4:** Pattern matrix table. Relationship between the correlation coefficients of sensory attributes with the three major components’ axes.

	Component			Communalities
	1	2	3	
**Color**	**0.713**	0.332	−0.208	0.747
**Porosity**	**0.877**	0.042	0.098	0.741
**Aroma**	−0.101	−0.143	**−0.875**	0.746
**Sweetness**	0.106	0.127	**−0.773**	0.672
**Softness**	**−0.739**	0.380	0.037	0.693
**Brittleness**	−0.012	**0.921**	0.036	0.848

Extraction Method: Principal Component Analysis. Rotation Method: Oblimin with Kaiser Normalization. Bold font: Coefficients taken into account when sensory attributes were associated with components.

**Table 5:** Identification, odor description, odor threshold, concentration (mg/kg of sample) and Odor Activity Values (in parentheses) of volatiles detected in different cake samples.

Peak No *^a^*	Compound *^b^*	RI *^c^*	ID *^d^*	Odor Description *^e^*	OT *^f^*(mg/kg)	Concentration (OAV)			
						Carob		Cocoa	
	*Aldehydes (18)*								
**1**	**3-Methyl-butanal**	911	1	Malty, chocolate, cocoa	0.0002 ^1^	357.4 ± 42.8	**(1786.8)**	368.3 ± 77.1	**(1841.4)**
**3**	**Pentanal**	971	1	Almond, pungent	0.012 ^1^	525.2 ± 91.8	**(43.8)**	520.9 ± 88.1	**(43.4)**
**7**	**Hexanal**	1069	1,2	Grass, fatty	0.0045 ^2^	1453 ± 89.9	**(323.0)**	1400 ± 278.1	**(311.1)**
8	(*E*)-2-Pentenal	1122	1	Apple, fruity, pungent	0.31 ^3^	199.1 ± 22.5	(0.6)	− *^g^*	
**12**	**Heptanal**	1178	1	Fatty, rancid	0.003 ^1^	294.7 ± 37.9 *	**(98.2)**	212.1 ± 21.8	**(70.7)**
**15**	**(*E*)-2-Hexenal**	1211	1,2	Green, apple	0.017 ^4^	113 ± 17.1 *	**(6.6)**	37.5 ± 6.1	**(2.2)**
**22**	**Octanal**	1282	1	Citrus, green, oily	0.007 ^4^	295.8 ± 36.7 *	**(422.6)**	217.9 ± 29.4	**(311.3)**
**25**	**(*E*)-2-Heptenal**	1314	1	Green, fatty	0.013 ^4^	217.2 ± 19.0	**(16.7)**	229.0 ± 36.4	**(10.5)**
**34**	**Nonanal**	1386	1	Tallowy, soapy	0.001 ^1^	358.4 ± 24.9	**(358.4)**	309.7 ± 17.6	**(309.7)**
**40**	**(*E*)-2-Octenal**	1418	1	Fresh, leafy, fatty	0.003 ^4^	183.7 ± 8.8 *	**(61.2)**	128.5 ± 7.9	**(42.8)**
**51**	**(*E,E*)-2,4-Heptadienal**	1484	1	Nutty, fatty, hay, fishy	0.0154 ^5^	228.2 ± 25.0	**(14.8)**	214.8 ± 17.6	**(13.9)**
**56**	**Benzaldehyde**	1508	1	Bitter almond	0.35 ^1^	620.3 ± 23.6 *	**(1.8)**	1623.2 ± 182.7	**(4.6)**
**58**	**(*E*)-2-Nonenal**	1522	1	Cucumber, fatty	0.00008 ^1^	114.9 ± 9.0 *	**(1436.3)**	75.0 ± 10.6	**(937.5)**
**68**	**Phenylacetaldehyde**	1626	1	Honey, green	0.004 ^1^	138.2 ± 13.9 *	**(34.6)**	407.7 ± 20.4	**(101.9)**
**77**	**Dodecanal**	1701	1	Lily, fat, citrus	0.0016 ^3^	60.5 ± 6.7 *	**(37.8)**	37.9 ± 1.0	**(23.7)**
**82**	**(*E,Z*)-2,4-Decadienal**	1754	1	Fried, fatty	0.00007 ^1^	69.1 ± 5.8c *	**(987.1)**	49.3 ± 2.4	**(704.3)**
**85**	**(*E,E*)-2,4-Decadienal**	1794	1	Fatty, fried, citrus	0.00007 ^1^	216.9 ± 4.9 *	**(3098.6)**	173.1 ± 15.5	**(2472.9)**
113	Vanillin	2542	1,2	Vanilla, chocolate	0.058 ^1^	5.3 ± 0.3	(0.1)	6.1 ± 2.8	(0.1)
	**Subtotal**					**5508.5**	**(8728.9)**	5917.9	**(7202.1)**
	*Alcohols (17)*								
2	Ethyl alcohol	925	1	Pleasant, fresh	100 ^2^	4351 ± 610.3	(<0.1)	3734.7 ± 381	(<0.1)
9	1-Butanol	1142	1	Fruity	0.50 ^4^	48.0 ± 3.4	(0.1)	42.6 ± 13.1	(0.1)
10	1-Penten-3-ol	1159	1	Butter, pungent	0.40 ^4^	73.4 ± 19.4	(0.2)	103.1 ± 27.2	(0.3)
**17**	**1-Pentanol**	1249	1,2	Floral, sweet, green	0.1502 ^5^	441.3 ± 89.2	**(2.9)**	568.8 ± 99.9	**(3.8)**
27	(*Z*)-2-Penten-1-ol	1321	1	Fruity, green	0.72 ^3^	163.1 ± 24.3 *	(0.2)	232.1 ± 28.0	(0.3)
**31**	**1-Hexanol**	1353	1,2	Green, fatty, leafy	0.5 ^2^	1602 ± 125.2	**(3.2)**	1800.8 ± 263.7	**(3.6** **)**
37	2-Butoxy-ethanol	1394	1	Earthy, nutty	0.88 ^3^	174.9 ± 8.0 *	(0.2)	242.8 ± 17.5	(0.3)
**45**	**1-Octen-3-ol**	1452	1	Earthy, green	0.0014 ^1^	102.1 ± 14.5 *	**(72.9)**	146.1 ± 26.5	**(104.4)**
**47**	**1-Heptanol**	1456	1	Green, fresh, nutty	0.425 ^2^	429.2 ± 53.7 *	**(1.0)**	531.0 ± 31.7	**(1.2)**
53	2-Ethyl-1-hexanol	1491	1	Green, vegetable	25.48 ^5^	263.6 ± 23.3	(0.9)	263.2 ± 26.2	(0.9)
**60**	**1-Octanol**	1557	1,2	Herbal, fatty, green	0.1258 ^5^	269.8 ± 20.4	**(2.1)**	249.9 ± 7.5	**(2.0)**
**67**	**(*E*)-2-Octen-1-ol**	1612	1	Green, citrus	0.02 ^3^	28.2 ± 1.4	**(1.4)**	40.6 ± 10.2	**(2.0)**
**71**	**1-Nonanol**	1659	1	Oily, green, floral	0.0455 ^5^	94.8 ± 10.1	**(2.1)**	90.6 ± 2.2	**(2.0)**
89	Benzyl alcohol	1866	1	Sweet, fresh	2.54 ^5^	314.1 ± 12.7	(0.1)	316.9 ± 22.0	(0.1)
91	Phenylethyl alcohol	1897	1,2	Floral	0.57 ^5^	100.3 ± 11.2 *	(0.2)	314.3 ± 13.4	(0.6)
96	1-Dodecanol	1965	1	Sweet, soapy, waxy	0.073 ^3^	61.7 ± 16.0 *	(0.8)	28.5 ± 1.3	(0.4)
**104**	**2-Methoxy-4-vinylphenol**	2183	1	Herbal	0.003 ^1^	17.5 ± 1.6 *	(5.8)	11.0 ± 1.6	(3.7)
	**Subtotal**					**8534.8**	**(92.7)**	**8717.0**	**(124.1)**
	*Ketones (12)*								
**4**	**1-Penten-3-one**	1015	1	Pungent, spicy	0.0013 ^3^	147.9 ± 13.3 *	**(113.8)**	106.1 ± 18.1	**(81.6)**
**5**	**2,3-Pentanedione**	1051	1	Caramel, buttery	0.020 ^1^	135.8 ± 19.1 *	**(6.8)**	97.3 ± 13.8	**(4.9)**
**11**	**2-Heptanone**	1175	1,2	Fruity, green	0.14 ^4^	233.2 ± 32.5 *	**(1.7)**	132 ± 6.4	(0.9)
20	3-Hydroxy-2-butanone	1276	1,2	Fatty, creamy	0.8 ^3^	−		38.6 ± 4.3	(<0.1)
**21**	**2-Octanone**	1278	1	Earthy, herbal	0.05 ^4^	54.1 ± 5.2	**(1.1)**	40.5 ± 8.2	(0.8)
23	1-Hydroxy-2-propanone	1292	1	Caramel, sweet	100 ^4^	305.1 ± 36.6	(<0.1)	−	
**28**	**6-Methyl-5-hepten-2-one**	1319	1	Earthy, fruity	0.068 ^5^	112.5 ± 17.1 *	**(1.7)**	59.9 ± 4.9	(0.9)
**33**	**2-Nonanone**	1381	1	Floral, fatty	0.005 ^1^	199.1 ± 5.9 *	**(39.8)**	70.9 ± 7.8	**(14.2)**
52	2-Decanone	1486	1	Orange, floral, fatty		tr *^h^*		tr	
**57**	**3,5-Octadien-2-one**	1512	1	Fruity, fatty, mushroom	0.15 ^1^	419.8 ± 24.9 *	**(2.8)**	681.9 ± 49.3	**(4.5)**
**63**	**2-Undecanone**	1587	1	Fruity, fresh	0.007 ^1^	35.3 ± 2.1 *	**(5.0)**	25.9 ± 2.9	**(3.7)**
70	Acetophenone	1630	1	Floral, almond	0.065 ^4^	17.7 ± 4.6	(0.3)	25.1 ± 4.1	(0.4)
	**Subtotal**					**1660.5**	**(172.9)**	**1278.2**	**(112.0)**
	*Monocarbonic acids (16)*								
46	Acetic acid	1455	1,2	Pungent, sour	22 ^4^	234.0 ± 56.9	(<0.1)	tr	(<0.1)
59	Propanoic acid	1539		Rancid	2 ^4^	81.7 ± 13.6 *	(<0.1)	18.7 ± 2.6	(<0.1)
**61**	**2-Methylpropanoic acid**	1564	1	Rancid	0.05 ^4^	222.8 ± 29.0	**(4.5)**	−	
68	**Butanoic acid**	1624	1,2	Sweaty, cheesy	0.24 ^4^	557.0 ± 96.8	**(2.3)**	−	
73	2-Methyl-butanoic acid	1666	1	Acidic, cheesy	0.5 ^3^	162.6 ± 14.2 *	(0.3)	86.5 ± 4.2	(0.2)
79	Pentanoic acid	1736	1	Sweaty, acidic	3 ^3^	154.1 ± 13.6 *	(0.1)	11.3 ± 3.4	(<0.1)
86	4-Methyl-pentanoic acid	1799	1	Pungent, cheesy	0.81 ^3^	45.8 ± 4.5	0.1	−	
**88**	**Hexanoic acid**	1842	1,2	Rancid, sweaty	3 ^2^	6067 ± 310.7 *	**(2.0)**	968.3 ± 175.5	**(0.3)**
92	2-Ethylhexanoic acid	1943	1	Sweet, musty	27 ^3^	15.3 ± 2.1 *	(<0.1)	10.2 ± 1.9	(<0.1)
93	Heptanoic acid	1948	1	Rancid, sour	0.64 ^3^	57.9 ± 10.1 *	(0.1)	28.3 ± 1.2	(<0.1)
101	Octanoic acid	2055	1,2	Sweaty, fatty	3 ^2^	452 ± 261.8	(0.2)	55.2 ± 13.5	(<0.1)
102	Nonanoic acid	2162	1	Green, fatty	4.6 ^3^	4.2 ± 0.3	(<0.1)	4.2 ± 0.3	(<0.1)
107	Decanoic acid	2269	1	Fatty, rancid	10 ^3^	64.4 ± 9.9 *	(<0.1)	15.2 ± 1.9	(<0.1)
109	Benzoic acid	2417	1	Urine		31.7 ± 2.7 *		17.2 ± 6.7	
110	Dodecanoic acid	2482	1	Metallic, fatty		6.8 ± 0.9		−	
113	Hexadecanoic acid	2897	1	Waxy		24.5 ± 5.4		21.0 ± 4.1	
	**Subtotal**					**8182.2**	**(19.7)**	**1236.1**	**(2.2)**
	*Pyrazine/Pyrrole derivatives (19)*								
14	Pyrazine	1209	1	Sweet, nutty	75 ^1^	54.2 ± 9.0 *	(<0.1)	28.6 ± 9.3	(<0.1)
19	Methylpyrazine	1257	1	Cocoa, green, pop corn	60 ^3^	70.1 ± 7.1 *	(<0.1)	84.1 ± 8.5	(<0.1)
24	2,5-Dimethylpyrazine	1311	1	Chocolate, roasted nuts, earthy	1.7 ^4^	57.6 ± 14.3 *	(<0.1)	100.4 ± 4.8	(0.1)
26	2,6-Dimethylpyrazine	1317	1	Chocolate, roasted nuts, fried potato	1.5 ^4^	99.2 ± 3.3 *	(0.1)	136.0 ± 18.3	(0.1)
29	2,3-Dimethylpyrazine	1335	1	Nutty, cocoa, coffee	2.5 ^4^	38.5 ± 10.9 *	(<0.1)	224.5 ± 20.3	(0.1)
**32**	**2-Ethyl-6-methylpyrazine**	1373	1	Nutty, sweet, roasted	0.04 ^3^	36.0 ± 2.6 *	(0.9)	72.8 ± 6.1	**(1.8)**
35	2-Ethyl-3-methylpyrazine	1391	1	Potato, nutty, roasted, cereal	0.13 ^3^	54.9 ± 7.8	(0.4)	−	
**36**	**Trimethylpyrazine**	1391	1	Nutty, baked potato, roasted cocoa, burnt	0.023 ^4^	−		351.4 ± 31.3	**(15.3)**
**38**	**2-Methoxy-3-methyl pyrazine**	1398	1	Chocolate, nutty, earthy, roasted	0.004 ^3^	170.3 ± 12.7	**(42.6** **)**	165.5 ± 13.3	**(41.4)**
**41**	**3-Ethyl-2,5-dimethylpyrazine**	1433	1	Cocoa, coffee, roasted, nutty	0.0004 ^1^	25.8 ± 4.6 *	**(64.5** **)**	87.5 ± 10.4	**(218.8)**
42	2,5-Diethylpyrazine	1444	1	Cocoa, roasted, nutty	0.02 ^3^	−		9.9 ± 1.3	(0.5)
**44**	**2-Ethyl-3,5-dimethyl-pyrazine**	1448	1	Cocoa, nutty, roasted, woody	0.001 ^3^	−		100.5 ± 10.6	**(100.5)**
48	Tetramethylpyrazine	1462	1	Nutty, musty, chocolate	1 ^3^	tr	(<0.1)	370.1 ± 18.8	(0.4)
50	3,5-Diethyl-2-methylpyrazine	1481	1	Nutty, meaty vegetable		−		14.5 ± 6.0	
55	2,3,5-Trimethyl-6-ethylpyrazine	1502	1			−		141.9 ± 12.1	
64	1-Ethyl-1H-pyrrole-2-carboxaldehyde	1589	1	Roasted, burnt, smoky		57.3 ± 1.1		−	
74	2-Acetyl-6-methylpyrazine	1675	1	Roasted coffee, cocoa	3 ^3^	−		17.2 ± 1.6	(<0.1)
9	2-Acetylpyrrole	1957	1	Earthy, hazelnut	58.6 ^3^	1531 ± 57.7 *	(<0.1)	467 ± 44.1	(<0.1)
107	2-Formyl pyrrole	2006	1	Musty, beefy, coffee		85.5 ± 8.6 *		56.8 ± 8.6	
	**Subtotal**					**2280.4**	**(108.5)**	**2414.2**	**(378.8)**
	*Furan/Pyran derivatives (11)*								
**16**	**2-Pentyl furan**	1226	1	Green, earthy, beany	**0.006** ^5^	212.2 ± 17.4 *	**(35.4)**	116 ± 27.2	**(19.3)**
18	Dihydro-2-methyl-3(*2H*)-furanone	1257	1	Sweet, caramel, nutty	−	128.4 ± 20.7 *	−	64.6 ± 2.7	−
**49**	**Furfural**	1464	1	Sweet, bready, almond, caramel, fruity	3 ^2^	4865 ± 346.2 *	**(1.6)**	503.2 ± 14.7	(0.2)
54	2-Acetylfuran	1499	1	Balsamic, cinnamon, sweet, cocoa	10 ^1^	630.4 ± 28.4 *	(0.1)	65.7 ± 3.2	(<0.1)
62	5-Methyl-furfural	1565	1	Raw potato, sweet, grass, almond	0.5 ^3^	116.4 ± 19.6 *	(0.2)	241.5 ± 44.0	(0.5)
72	2-Furanmethanol	1663	1	Burnt sugar, bread, coffee	2 ^4^	1213 ± 15.6 *	(0.6)	358.4 ± 7.7	(0.2)
78	5-Methyl-2-furanmethanol	1725	1	Sweet, caramel		90.6 ± 2.6 *		22.5 ± 2.5	
95	Maltol	1948	1	Caramel	2.5 ^4^	62.9 ± 9.6 *	(<0.1)	38.8 ± 4.0	(<0.1)
107	2,3-dihydro-3.5-dihydroxy-6-methyl-4H-pyran-4-one	2249	1	Caramel, roasted		−		20.3 ± 5.0	
109	3,5-Dihydroxy-2-methyl-4H-pyran-4-one (5-hydroxymaltol)	2282	1	Caramel, roasted		22.6 ± 3.3		−	
112	5-Hydroxymethylfurfural	2494	1	Fatty, buttery, caramel	1000 ^3^	43.9 ± 12.2 *	(<0.1)	4.8 ± 0.5	(<0.1)
	**Subtotal**					**7385.2**	**(37.9** **)**	**1435.8**	**(20.2)**
	*Lactones (10)*								
39	5-Methyl-2(3H)furanone (*α*-Angelica lactone)	1403	1	Coconut, nutty, herbal, sweet		90.8 ± 1.3		−	
66	Dihydro-2(3H)-furanone (Butyrolactone)	1605	1	Caramel, buttery	1 ^4^	75.5 ± 5.4	(0.1)	67.5 ± 0.7	(0.1)
**83**	**5-Ethyldihydro-2(3H)-furanone** (*γ*-Hexalactone)	1679	1	Sweet, herbal, coconut	0.05 ^3^	70.3 ± 6.0 *	**(1.4)**	46.5 ± 2.7	**(0.9)**
80	2(*5H*)-Furanone(γ-Crotonolactone)	1738	1	Buttery		16.9 ± 2.0 *	−	5.7 ± 0.7	−
75	Tetrahydro-6-methyl-2H-pyran-2-one *(δ*-Hexalactone)	1764	1	Creamy, fruity, coconut		32.5 ± 0.4		−	
**90**	**5-Butyldihydro-2(*3H*) furanone** (*γ*-Octalactone)	1890	1	Sweet, creamy, dairy, coconut	0.007 ^4^	26.8 ± 4.3 *	**(3.8)**	11.5 ± 1.7	**(1.6)**
**98**	**Dihydro-5-pentyl-2(3H)-furanone** (*γ*-Nonalactone)	2000	1	Coconut, sweet, creamy, fatty	0.0097 ^3^	33.5 ± 3.3 *	**(3.5)**	15.4 ± 0.7	**(1.6)**
101	5-Acetyldihydro-2(3H)furanone (Solerone)	2047	1	Wine-like, fruity		24.7 ± 0.2		−	
**104**	**Tetrahydro-6-pentyl-2H-pyran-2-one** (*δ*-Decalactone)	2162	1	Sweet, coconut, creamy, peach	0.031 ^3^	127.5 ± 7.2 *	**(4.1)**	67.0 ± 4.0	**(2.2)**
106	5,6-Dihydro-6-pentyl-2H-pyran-2-one (*δ-*Decenolactone)	2200	1	Coconut, creamy, peach, herbal		31.1 ± 2.6		15.5 ± 6.7	
	**Subtotal**					**529.6**	**(13.2** **)**	**229.1**	**(6.4)**
	*Esters (6)*								
30	Ethyl 2-hydroxypropanoate	1342	1	Sweet, fruity, buttery	50 ^3^	35.1 ± 2.7	(<0.1)	44.2 ± 8.6	(0.0)
65	Methyl benzoate	1602	1	Dry plum, sweet, herbal	0.073 ^3^	45.8 ± 3.8	(0.6)	−	
81	Methyl phenylacetate	1749	1	Honey, floral, fruity		−		5.7 ± 1.3	
84	Ethyl phenylacetate	1775	1	Floral, honey, cocoa		−		6.2 ± 1.5	
87	2-Phenylethyl acetate	1801	1,2	Floral, rose, honey	0.25 ^5^	−		43.1 ± 2.8	(0.2)
100	Isopropyl myristate	2032	1	Fatty		33.4 ± 12.0 *		10.5 ± 2.0	
	**Subtotal**					**114.3**	**(0.6** **)**	**109.7**	**(2.1)**
	*Sulfur compounds (2)*								
**6**	**Dimethyl disulfide**	1058	1	Rotten, cabbage	0.012 ^4^	127.2 ± 5.2	**(10.6** **)**	−	
**43**	**3-(Methylthio)-propanal** (Methional)	1448	1	Cooked potatoes	0.0002 ^1^	35.8 ± 2.0	**(179.0** **)**	−	
	Subtotal					**163.0**	**(189.6** **)**		
	*Terpenes (2)*								
**13**	***D*-Limonene**	1182	1,2	Citrus	0.3 ^4^	207.8 ± 27.7	(0.7)	184 ± 16.1	(0.6)
76	*α*-Terpineol	1686	1	Fruity, floral	0.28 ^3^	76.1 ± 7.2	(0.3)	62.9 ± 6.3	(0.2)
	**Subtotal**					**283.9**	**(1.0** **)**	**246.9**	**(0.8** **)**
	**Total**					**34585.2**	**(9365.2)**	**21585.0**	**(7848.6)**

*^a^* Compound numbers according to the order of elution. In bold are the compounds having OAV > 1 in at least one cake sample. *^b^* Retention index (RI) on the capillary DB-Wax. *^c^* Mean values (±SD) (*n* = 4). Means in the same row noted with an asterisk are significantly different (*p* < 0.05) as determined by Independent samples *t*-Test. *^d^* ID identification based on agreement with: 1, MS data stored in NIST library (v2.0f, 2008, Gaithersburg, MD, USA) and published RIs (http://www.pherobase.com/ [36]; http://www.flavornet.org/ [37]; the literature data); 2, MS data and RI of standard compounds. *^e^* Odor descriptions originate from Flavornet [37], Pherobase [36] and Good Scents Company Information System (www.thegoodscentscompany.com/ [38]) online databases. *^f^* Threshold values in water were taken from the following literature sources: *^1^* Buttery and Ling [39]; *^2^* Pino and Mesa [40]; *^3^* van Gemert [41]; *^4^* Buttery and Ling [42]; *^5^* Giri et al. [43]. *^g^* −: Not detected. *^h^* tr: Compounds detected in the headspace of the samples but not quantified.
